# The Shape of μ—How Morphological Analyses Shape the Study of Microglia

**DOI:** 10.3389/fncel.2022.942462

**Published:** 2022-06-29

**Authors:** Lance Fredrick Pahutan Bosch, Katrin Kierdorf

**Affiliations:** ^1^Institute of Neuropathology, Faculty of Medicine, University of Freiburg, Freiburg, Germany; ^2^Faculty of Biology, University of Freiburg, Freiburg, Germany; ^3^Center for Basics in NeuroModulation (NeuroModulBasics), Faculty of Medicine, University of Freiburg, Freiburg, Germany; ^4^CIBSS–Centre for Integrative Biological Signalling Studies, University of Freiburg, Freiburg, Germany

**Keywords:** microglia, microglia morphology, 3D reconstruction, *in vivo* imaging, electron microscopy, automated image analysis

## Abstract

Microglia, the innate immune cells of the CNS parenchyma, serve as the first line of defense in a myriad of neurodevelopmental, neurodegenerative, and neuroinflammatory conditions. In response to the peripheral inflammation, circulating mediators, and other external signals that are produced by these conditions, microglia dynamically employ different transcriptional programs as well as morphological adaptations to maintain homeostasis. To understand these cells’ function, the field has established a number of essential analysis approaches, such as gene expression, cell quantification, and morphological reconstruction. Although high-throughput approaches are becoming commonplace in regard to other types of analyses (e.g., single-cell scRNA-seq), a similar standard for morphological reconstruction has yet to be established. In this review, we offer an overview of microglial morphological analysis methods, exploring the advantages and disadvantages of each, highlighting a number of key studies, and emphasizing how morphological analysis has significantly contributed to our understanding of microglial function in the CNS parenchyma. In doing so, we advocate for the use of unbiased, automated morphological reconstruction approaches in future studies, in order to capitalize on the valuable information embedded in the cellular structures microglia inhabit.

## Introduction

Microglia are the central force of the central nervous system’s (CNS) innate immune system, serving as the first parenchymal respondents in a multitude of neurodevelopmental, neurodegenerative, and neuroinflammatory diseases (Kierdorf and Prinz, 2017; Li and Barres, 2018). Emerging from embryonic progenitors during development, microglia differentiate into specialized tissue-resident macrophages possessing small immotile cell somas surrounded by elongated, ramified processes that are constantly surveying the surrounding CNS parenchyma (Davalos et al., 2005; Nimmerjahn et al., 2005). There is little to no overlap in the parenchymal volumes monitored by individual microglia, resulting in a grid-like system that efficiently and effectively guards the CNS (Kettenmann et al., 2011). Upon insult, however, this system is disrupted. Microglia undergo rapid morphological transitions: from their ramified, homeostatic form; to a hypertrophic state; and ultimately to motile, amoeboid shapes. Those in the immediate vicinity of an insulting stimuli then become activated, rapidly migrate toward the insult, proliferate, and release inflammation-resolving cytokines. Upon resolution, these cells return to their ramified states and resume their homeostatic network behaviors (Roufagalas et al., 2021). Although the activation-induced adjustment to an amoeboid state is a well-known activation form, microglia can exhibit a wide range of morphologies depending on the insult or disease condition (Erny et al., 2015; Savage et al., 2019; Lopez-Rodriguez et al., 2021; van Olst et al., 2021). Indeed, this morphological plasticity is a defining feature of microglia, and has been noted as such since their discovery by Del Rio-Hortega (1919, 1932).

Recent advances in high-throughput, single cell transcriptomic profiling allowed for the definition of certain genes as markers for microglial activation states: the homeostatic markers *Transmembrane protein 119* (*Tmem119*) and purinergic receptor *P2ry12* (Bennett et al., 2016; van Wageningen et al., 2019); as well as the neurodegeneration-associated markers *Triggering receptor expressed on myeloid cells 2* (*Trem2*) and *Apolipoprotein E* (*ApoE*) (Keren-Shaul et al., 2017; Krasemann et al., 2017). Meanwhile, links between morphological states and distinct activation states are yet to be similarly defined. Moreover, though morphological reconstruction is an essential method of analyzing microglial adaptation in response to inflammation, released mediators, and disease conditions in many studies (Savage et al., 2019), it is rarely employed in the high-throughput manner that has become commonplace for gene expression analyses (e.g., scRNA-seq). This is especially interesting given that many morphological characteristics of microglia appear to be evolutionarily conserved across species—unlike astrocytes, for example (Oberheim et al., 2009). Studies have shown that although murine microglia may exhibit a more heterogeneous range of cell-body shapes than that of humans, many of their characteristics remain consistent, including size (mouse vs. human) (Torres-Platas et al., 2014) or number of primary processes (rat vs. human) (Kongsui et al., 2014). Consequently, high-throughput morphological analyses of microglia in animal models have the prospect of generating pathology-relevant knowledge that is potentially translatable to humans.

Here, we provide a summary of the techniques available in microglial morphological analyses, as well as their evolution over time. We further discuss the advantages and disadvantages of each technique, highlighting some key studies that have expanded our comprehension of microglia. Simultaneously, we emphasize the need for automated, high-throughput reconstruction approaches in future studies to further extract the valuable information that lie in the unique structures microglia inhabit.

## Assessment of Microglial Morphology on Histological Sections (2D)

Since their debut description by Pío del Río-Hortega, the morphology of microglia has been key to understanding their function (Del Rio-Hortega, 1919). Through a silver carbonate staining protocol, microglia-specific staining in 20 μm sections (Del Rio-Hortega, 1919, 1932) could be achieved, revealing: their small cell somas; their arboreal protrusions; and their ability to transmute themselves in response to specific stimuli, such as the already earlier described phagocytic “*Stäbchenzellen*” or engulfing “*granuloadipose bodies*” (Nissl, 1899; Achúcarro, 1909). These features were recorded in hand-drawn depictions following observation under simple light microscopes.

Though the options have widened over the last century, 2D morphological reconstructions of microglia have continued to be a relevant method of analysis in the field. To date, microglia are labeled by either antibodies against markers such as ionized binding adaptor protein 1 (Iba-1) in immunohistochemistry or immunofluorescence staining, or by transgenic reporter models such as the *Cx3cr1^GFP^* mouse model (Jung et al., 2000), imaged, and subsequently analyzed. Here, thin sections, similar to those first used by del Río-Hortega, are preferred to reduce the possibility of capturing overlapping cells. Initially, studies employing this style of morphological analysis simply showcased representative images of microglia in the analyzed conditions, as the difference between the homeostatic and activated cells was considered to be self-evident (Ayoub and Salm, 2003; Mildner et al., 2011; Streit et al., 2018). Later, these representative images were often paired with quantifications to enumerate the extent to which a particular genetic perturbation, disease condition, or intervention activates the microglial compartment; manual measurements of parameters like branch length and cell soma size are also used to highlight subtle changes (Torres-Platas et al., 2014). To mitigate analyzer subjectivity, morphologic assessments can be accompanied by descriptions of the cells’ anatomical localization and expression of certain surface markers; for example, the correlations shown to exist between amoeboid microglia and amyloid-beta plaques (Plescher et al., 2018).

Currently, automated approaches are being developed, improving the reproducibility of results as well as allowing more complex parameters to be measured from the same 2D imaging data. In these approaches, 2D images undergo a binarization *via* signal-thresholding (Zanier et al., 2015), which can then be further quality controlled (e.g., branch-joining) (Fernández-Arjona et al., 2017). Following this, software such as ImageJ can calculate parameters such as fractal dimension, lacunarity, and convex hull area (Orłowski et al., 2003; Karperien et al., 2013; Karperien and Jelinek, 2015; Fernández-Arjona et al., 2017), which can then be used to assess the structural complexity of a given microglia (Verdonk et al., 2016). Moreover, the binarized signal can be reinterpreted into a 2D cell skeleton, allowing for other complexity calculations (Soltys et al., 2005). Through these means, microglial morphology can be evaluated along multiple axes (e.g., principal component analysis (PCA), hierarchical clustering) (Verdonk et al., 2016; Fernández-Arjona et al., 2017), and ergo, rigid categories such as ramified and amoeboid can be replaced by more nuanced, value-derived categories.

**FIGURE 1:**
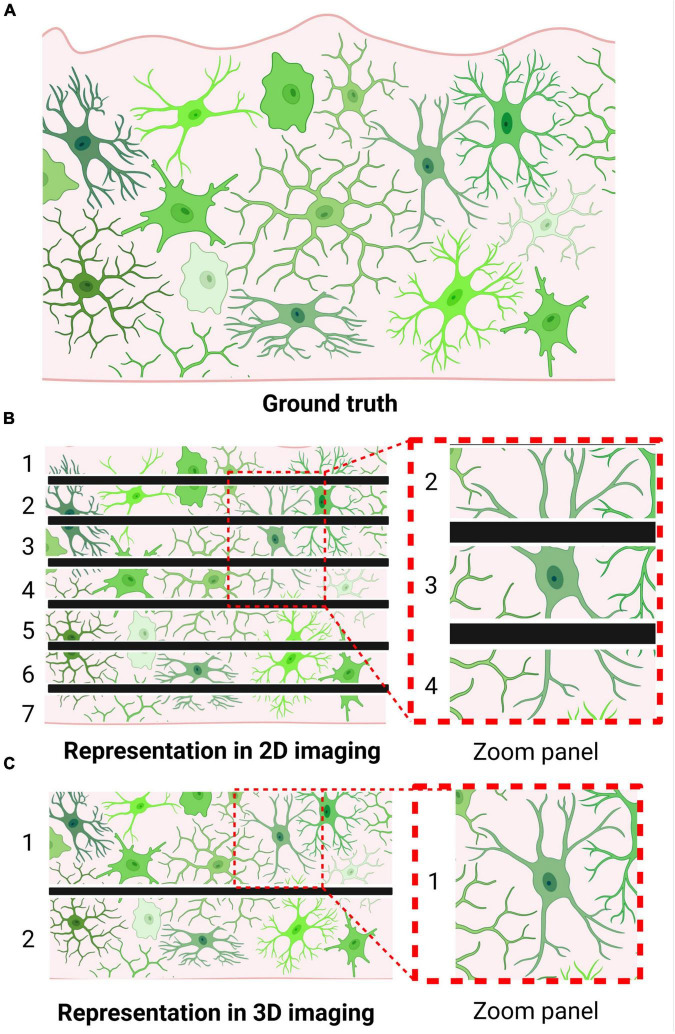
Microglia may be morphologically misrepresented depending on imaging technique. **(A)** Brain tissue containing labeled microglia (i.e., visualization of ground truth). **(B)** Visualization of microglial morphology following thin sectioning, as required by 2D imaging approaches. Numbers indicate the number of sections produced; black bars indicate borders of obtained tissue sections. Zoom panel showcases a cell that could be misclassified as an activated microglia due to the method of sectioning. **(C)** Visualization of microglial morphology following thick sectioning, as permitted by 3D imaging approaches. Numbers indicate the number of sections produced; black bars indicate borders of obtained tissue sections. Zoom panel showcases the same cell as in panel **(B)**; however, due to the thicker sectioning, its bipolar branch structure can be accurately represented.

The simplicity of this popular method of morphological analysis is also the source of its major limitations. The thin sections are not likely to capture a microglia’s entire structure within frame, meaning unique structures may be misrepresented in the XY plane (Figure 1) (Taylor et al., 2014). Thus, regardless of the fact that 2D morphological assessments on histological sections constitute a simple yet robust tool for microglial analysis, appropriate measures must be taken to prevent data misinterpretation.

## 3D Rendering of Microglial Morphology *Via* Serial Electron Microscopy

Microglia were first visualized within the rat parietal cortex *via* transmission electron microscopy (TEM) in the late 1950’s (Schultz et al., 1957). In these images, microglia could be identified by ultrastructural morphological features such as: nuclear membrane-associated electron-dense heterochromatin; small, bean-shaped nuclei; and a thin cell soma containing lysosomes, vesicles, and mitochondria (Mori and Leblond, 1969; Shapiro et al., 2009). Given the high subcellular resolution of these images, intracellular functional changes can be studied, such as phagocytosis, mitochondrial dysfunction, proliferation, and apoptosis. Intercellular changes can also be tracked; somatic microglia-neuron junctions were recently demonstrated by the use of this technique (Cserép et al., 2020, 2021). With the identification of microglia being further simplified *via* Immunogold staining, EM is a reliable tool in diagnosing microglial function in the healthy and diseased CNS in a variety of species (Savage et al., 2019; Császár et al., 2022).

In spite of these advantages, the ultrathin sections required for EM limit its usefulness in analyzing microglial morphological features in 2D. However, this shortcoming was bypassed through the development of serial sectioning electron microscopy (SSEM)—where EM is performed on consecutive sections—allowing researchers to create semi-3D reconstructions with the same level of ultrastructural detail. Nonetheless, SSEM can only join a limited number of sections and cannot always reconstruct ramified microglial cells in their entirety (Stalder et al., 2001; Tremblay et al., 2010). Recently this has been achieved in the murine hippocampus using serial block face scanning electron microscopy (SBEM) (Bolasco et al., 2018). This SBEM study offered a unique view on microglial morphology in the steady-state CNS: not only could features such as filopodia be very exactly measured, but also subcellular features such as organelles could be precisely measured concurrently (Bolasco et al., 2018). Still, both SSEM and SBEM require a high level of EM imaging expertise, time-intensive work, and expensive equipment, therefore making EM difficult to utilize without access to the necessary facilities. The large data sets generated by EM approaches are also not yet adequately addressed by these technical developments. Nevertheless, SSEM- and SBEM-based 3D reconstructions remain a powerful tool to explore microglial morphological changes at a subcellular resolution, making it ideal for immunometabolic or ultrastructural interaction studies.

## Analyzing Microglial Morphology *In Vivo*

In contrast to imaging brain sections, *in vivo* imaging provides the possibility of recording the morphological shifts of microglia over time, as well as the dynamics of those shifts. A common method to achieve *in vivo* imaging is through the creation of an acute or chronic skull window in microglial reporter mice, such as *Cx3cr1^GFP^* mice (Davalos et al., 2005; Nimmerjahn et al., 2005; Meyer-Luehmann et al., 2008). This skull window can consist of an area of thinned bone (Davalos et al., 2005; Nimmerjahn et al., 2005; Yang et al., 2010), a skull area replaced with a glass coverslip (Levasseur et al., 1975; Meyer-Luehmann et al., 2008; Holtmaat et al., 2009), optically cleared bone (Zhao et al., 2018), or other variants (Portera-Cailliau et al., 2005; Drew et al., 2010; Nimmerjahn, 2012; Shih et al., 2012; Cruz-Martin and Portera-Cailliau, 2014; Dombeck and Tank, 2014; Dorand et al., 2014; Goldey et al., 2014; Roome and Kuhn, 2014; Takehara et al., 2014; Heo et al., 2016; Kim et al., 2016). Through this window, a two photon microscope is able to excite the expressed fluorescent proteins, visualize microglia within the range of its camera system, and record their behavior over time. With this spatiotemporally sensitive technique, the dynamic nature of microglial morphology can be appreciated in a way that is impossible in static images.

As opposed to their description as “resting” based on their appearance in static images, *in vivo* imaging revealed that steady-state microglia are anything but at rest (Streit et al., 1988). Rather, adult microglia were found constantly surveying their surroundings, extending and retracting processes at an average rate of 1.5 μm per minute (Nimmerjahn et al., 2005; Fuhrmann et al., 2010). Moreover, adjacent microglia can initiate a targeted movement of their processes to respond to potential tissue damage, performing homeostasis-restoring functions such as the isolation of lesioned blood vessels or removal of dead cells in laser-induced brain lesions (Davalos et al., 2005; Nimmerjahn et al., 2005; d’Errico et al., 2022). This monitoring of the functional response of microglia and how it relates to their morphological state has also been performed over longer time windows, allowing for their description over several months during healthy aging as well as in Alzheimer’s disease mouse models (Hefendehl et al., 2014; Füger et al., 2017). These morphological and behavioral changes can be represented as dose-response curves in relation to a given stimuli (e.g., microglial translocation vs. days of Csf1r inhibitor treatment), therefore measuring parameters that cannot be calculated with precision in static images (Davalos et al., 2005; Mendes et al., 2021).

Whilst *in vivo* analyses play a crucial role in deciphering microglial function, the surgical procedure required to collect the imaging data introduces restrictions and artifacts pertinent to this method alone. First, the skull window can potentially cause localized inflammation in the CNS parenchyma, creating technical artifacts in the data (Hefendehl et al., 2011; Zhao et al., 2018). In addition, as the method can only image at a depth relative to the skull window created (e.g., 500–1,000 μm from the pial surface), certain CNS regions are barred from an *in vivo* analysis approach (Hierro-Bujalance et al., 2018). Second, the technique relies on the labeling of cells *via* fluorescent reporters or the injection of antibodies or dyes. As microglia are tough to reach with antibodies and dyes, the ability to explore the morphological adaptations of specific microglial subpopulations tends to be reliant on the availability of suitable transgenic reporter lines. This reliance has the potential to produce artifacts: in *Cx3cr1^GFP^* mice—a commonly used reporter line—the knock-in of the transgenic eGFP into the *Cx3cr1* locus has been reported to alter the morphological and behavioral dynamics of microglia in select contexts (Kierdorf et al., 2013; Lowery et al., 2017). Additionally, whether a particular experimental condition affects the fluorescence of the labeled cells also needs to be taken into consideration. For example, laser ablation can cause a loss of EGFP fluorescence within a 20 μm radius in *Cx3cr1^GFP^* mice (Davalos et al., 2005). Third, the time scale used in an *in vivo* imaging analysis can significantly affect the amount of data processing required: a day’s worth of imaging data assessed by hour results in 24 analyzable data points, whereas analyzing by minute results in 2880 analyzable data points. How to interpret the recorded morphological shifts is a similarly important consideration. Calculating the movement of all microglial processes within a given radius (Davalos et al., 2005) is less time-intensive than recording the microglial response on a per-protrusion level (Nimmerjahn et al., 2005), though it lacks the ability to decipher response heterogeneity within the population. Conversely, interpreting the morphological shifts in fine detail often necessitates the analysis of a few representative cells, introducing selection bias. Thankfully, automated pipelines can reduce the laboriousness of analyzing *in vivo* data and therefore eliminate the need to compromise. Following a thresholding procedure, microglial response to a stimulus or condition can be interpreted by software through the use of user-defined thresholds in specific morphological parameters (e.g., cell soma size) (Kozlowski and Weimer, 2012; Bosco et al., 2015; Bloomfield et al., 2018). Even amorphous microglial states can be determined *in vivo via* the creation of indices that interpret shifts in multiple parameters simultaneously (e.g., an inflammation index) (Clarke et al., 2021). Moreover, other pipelines have been developed that are able to track the movements of single processes, allowing for the batch processing of *in vivo* imaging data without significant user intervention (Paris et al., 2018). As a result, these pipelines can be used in combination with advanced *in vivo* imaging setups to allow researchers to systematically interpret morphological changes longitudinally, for which there is no analytic equivalent.

## 3D Reconstruction of Microglial Morphology *Via* Confocal Microscopy

With the ongoing advances in confocal laser microscopy, the reconstruction of microglial structures in 3D can be considered to be the gold standard of morphological analyses. Applying many of the same principles used in 2D analysis, the mere addition of the Z dimension in 3D reconstructions allows the analyzer to process sections upwards of 100 μm, significantly reducing the possibility of misrepresenting a particular cell’s structure (Figure 1). With typical adult microglial cells arranging themselves at a consistent nuclei distance of approximately 60 μm (unpublished observation), these thick sections are able to capture a cell with all its processes. Thus, it is possible to accurately observe atypical microglial shapes that 2D analyses can struggle with (Savage et al., 2019). Furthermore, 3D imaging opens up the possibility of tracing individual microglial processes—from tip to root—in specialized programs such as IMARIS, permitting new morphological information to be acquired from these cells. These included parameters such as the number of terminal points, branch points, and number of cell segments (Goldmann et al., 2013; Kierdorf et al., 2013; Hagemeyer et al., 2016; Masuch et al., 2016). Moreover, these 3D representations allow for the quantification of parameters once arduous to decipher, such as cell volume and dendrite length, accurate to the nanometer (Erny et al., 2015). These advances allowed researchers to better evaluate microglial morphology not only as differences in kind (amoeboid or ramified, activated or homeostatic) but as differences in degree (retracted processes, reductions in branching complexity). Because of this ability to measure nuances, 3D reconstructions proved crucial in our understanding of the effects of environmental factors, disease conditions, or genetic perturbations, such as the influence of the host microbiota on microglial development, function, and maturation (Erny et al., 2015; Mezö et al., 2020; Mossad et al., 2022).

The benefits offered by 3D morphological reconstructions are not without caveats. Though a plethora of information can be extracted from a single reconstructed cell, the process of generating these models is often a tedious, time-intensive endeavor. The method is semi-automatic at best, often requiring manual editing of each reconstruction to represent cells faithfully. To compensate for this time investment, representative cells have to be chosen for each experimental condition, introducing the bias inherent in such selections. Worse still, this can result in morphological analyses that may include many biological replicates, experimental conditions, and timepoints, whilst ultimately representing the information embedded within a few dozen cells, risking the creation of a skewed dataset. Interestingly, though it addresses the limitations of 2D morphological analyses, the limitations of 3D reconstructions showcase why the two methods are often utilized in tandem: what is gained in fine detail is lost in an appreciation of the larger context, and vice versa.

There is, however, a growing number of resources available that enable reconstructions with minimal analyzer bias. Designed in programming languages such as C++(Xiao and Peng, 2013; Megjhani et al., 2015) and MATLAB (Heindl et al., 2018; York et al., 2018; Abdolhoseini et al., 2019), these published tools algorithmically reconstruct all the microglia captured in a given image *en masse*. These automated analysis pipelines therefore minimize both the time investment and the selection bias that were once inextricable from 3D reconstruction approaches, markedly improving the viability and scalability of the analysis. Furthermore, this facilitates the reconstruction of a greater number of microglia for every experimental condition of interest, resulting in datasets that exhibit the range of microglial morphologies that can occur simultaneously. An additional advantage is that many published algorithms are accompanied by thorough documentation, making it possible for the user to optimize the algorithms to their dataset. With each tool producing a customized set of data outputs, these automated options can enable researchers to analyze the specific morphological parameters that best address their research questions.

## Discussion

Given the volume of knowledge that has been ascertained about microglia through the use of the techniques described here, it is clear that morphological analysis will continue to be a mainstay within the field of neuroimmunology. By interpreting their structural adaptations in tandem with their transcriptomic profiles, it is possible to elucidate the heterogeneous and plastic nature of microglial function across development, health, and disease—as has been done in the research we have highlighted in this review. Yet, in an age of scientific publishing wherein single cell RNA sequencing of thousands of cells is considered to be a necessity, it is unfortunate that the morphological analysis of a meager amount of microglia can be considered sufficient. However, as we emphasized above, automated morphological analysis approaches are becoming more sophisticated and more accessible, and are designed for use in complement with new cutting-edge imaging techniques. These innovative approaches could soon become the new gold standard, ushering in an era wherein the morphological characteristics of whole microglial populations are efficiently evaluated with the depth previously reserved for single cells. Population-level datasets are also likely to provide insights into microglial function that could not be captured due to the technical limitations of the past. What insights into microglial function may specifically be gleaned remains to be seen, but what is certain is that our understanding of microglia is sure to expand through an increased utilization of these unbiased, high-throughput techniques.

## Author Contributions

LB wrote the manuscript and designed the figures. KK conceptualized, wrote, and edited this review. Both authors contributed to the article and approved the submitted version.

## Conflict of Interest

The authors declare that the research was conducted in the absence of any commercial or financial relationships that could be construed as a potential conflict of interest.

## Publisher’s Note

All claims expressed in this article are solely those of the authors and do not necessarily represent those of their affiliated organizations, or those of the publisher, the editors and the reviewers. Any product that may be evaluated in this article, or claim that may be made by its manufacturer, is not guaranteed or endorsed by the publisher.
